# Studies on the Structure and Properties of Membrane Phospholipase A_1_ Inclusion Bodies Formed at Low Growth Temperatures Using GFP Fusion Strategy

**DOI:** 10.3390/molecules26133936

**Published:** 2021-06-28

**Authors:** Svetlana I. Bakholdina, Anna M. Stenkova, Evgenia P. Bystritskaya, Evgeniy V. Sidorin, Natalya Yu. Kim, Ekaterina S. Menchinskaya, Tatiana Yu. Gorpenchenko, Dmitry L. Aminin, Nikita A. Shved, Tamara F. Solov’eva

**Affiliations:** 1 G.B. Elyakov Pacific Institute of Bioorganic Chemistry, Far-Eastern Branch of the Russian Academy of Sciences, Prospekt 100-let Vladivostoku 159, 690022 Vladivostok, Russia; belyjane@gmail.com (E.P.B.); sev1972@mail.ru (E.V.S.); natalya_kim@mail.ru (N.Y.K.); ekaterinamenchinskaya@gmail.com (E.S.M.); daminin@piboc.dvo.ru (D.L.A.); 2 Department of Medical Biology and Biotechnology, FEFU Campus, School of Biomedicine, Far Eastern Federal University, Russky Island Ajax Bay 10, 690922 Vladivostok, Russia; stenkova@gmail.com (A.M.S.); nikitawayfarer@yandex.ru (N.A.S.); 3 Federal Scientific Center of the East Asia Terrestrial Biodiversity, Far Eastern Branch of the Russian Academy of Sciences, Prospect 100-let Vladivostoku, 159, 690022 Vladivostok, Russia; gorpenchenko@biosoil.ru; 4 Department of Biomedical Science and Environmental Biology, Kaohsiung Medical University, 100, Shih-Chuan 1st Road, Kaohsiung 80708, Taiwan; 5 A.V. Zhirmunsky National Scientific Center of Marine Biology, Far Eastern Branch of the Russian Academy of Sciences, Palchevskogo str. 17, 690041 Vladivostok, Russia

**Keywords:** recombinant phospholipase A_1_, *Yersinia pseudotuberculosis*, inclusion bodies, fusion protein, green fluorescent protein

## Abstract

The effect of cultivation temperatures (37, 26, and 18 °C) on the conformational quality of *Yersinia pseudotuberculosis* phospholipase A_1_ (PldA) in inclusion bodies (IBs) was studied using green fluorescent protein (GFP) as a folding reporter. GFP was fused to the C-terminus of PldA to form the PldA-GFP chimeric protein. It was found that the maximum level of fluorescence and expression of the chimeric protein is observed in cells grown at 18 °C, while at 37 °C no formation of fluorescently active forms of PldA-GFP occurs. The size, stability in denaturant solutions, and enzymatic and biological activity of PldA-GFP IBs expressed at 18 °C, as well as the secondary structure and arrangement of protein molecules inside the IBs, were studied. Solubilization of the chimeric protein from IBs in urea and SDS is accompanied by its denaturation. The obtained data show the structural heterogeneity of PldA-GFP IBs. It can be assumed that compactly packed, properly folded, proteolytic resistant, and structurally less organized, susceptible to proteolysis polypeptides can coexist in PldA-GFP IBs. The use of GFP as a fusion partner improves the conformational quality of PldA, but negatively affects its enzymatic activity. The PldA-GFP IBs are not toxic to eukaryotic cells and have the property to penetrate neuroblastoma cells. Data presented in the work show that the GFP-marker can be useful not only as target protein folding indicator, but also as a tool for studying the molecular organization of IBs, their morphology, and localization in *E. coli*, as well as for visualization of IBs interactions with eukaryotic cells.

## 1. Introduction

The production of recombinant proteins in *Escherichia coli* is often accompanied by the formation of their insoluble aggregates, the so-called inclusion bodies (IBs), in the cell cytoplasm [1]. For a long time, it was believed that IBs consist of misfolded proteins, and the isolation of a recombinant protein from them in a functionally active form is a laborious and expensive procedure with a low yield [2]. However, it was later found that a protein in a native-like conformation can be present in IBs, and its amount can be controlled by manipulating the genetic and physiological parameters of expression [3]. A growing body of data confirms the fact that the protein within IBs adopts different conformational states ranging from unstructured, partly folded, native or native-like, to cross-β-sheet (amyloid-like) [4]. IBs containing a high percentage of properly folded protein can be functionally active and be used as nanomaterials in medicine and biotechnology [5]. The target protein from these IBs can be isolated under mild conditions without the use of renaturing steps and with high yield.

An important factor influencing the conformational quality of the protein in IBs is the expression temperature [6]. IBs synthesized at growth temperatures below the optimal 37 °C usually have a higher content of properly folded protein. The slower rate of protein production at low temperatures gives newly synthesized recombinant proteins more time to fold properly. Using low growth temperature, for a series of water-soluble globular proteins, including oligomeric ones, it was possible to obtain biologically active IBs with a high protein content in the native conformation, known as “nonclassical” IBs [7,8,9]. However, recently there was information that the globular protein L-asparaginase II forms “nonclassical” IBs, on the contrary, at elevated culture temperatures [10]. Moreover, it was reported that IBs formed at the optimal bacterial growth temperature contained a functionally active protein, and that the temperature regime did not affect the protein structure in IBs [11,12]. These facts demonstrate that the effect of the expression temperature on the conformational quality of IBs is ultimately determined by the nature of the protein, i.e., the intrinsic physicochemical properties of its amino acid sequence. At this time, the correlation between these factors affecting the structural organization of IBs is poorly understood. Most of the published research findings on this issue were performed on IBs of soluble globular proteins. IBs formed by membrane proteins are largely unexplored. In our work, the recombinant phospholipase A_1_ of *Yersinia pseudotuberculosis* as a model integral membrane protein was expressed in *E. coli* at different growth temperatures and the resulting IBs were characterized. In addition, determination of the growth temperature, which promotes the formation of IBs with a high content of correctly folded protein, can also be useful for developing novel, improved methods for biotechnology production of active enzyme. Lipolytic enzymes, including phospholipase A_1_, find wide application in food, cosmetic, and pharmaceutical industries [13].

The phospholipase A_1_ from the outer membrane protein of *Y. pseudotuberculosis* (PldA—detergent-resistant phospholipase A_1_, EC 3.1.1.32) is a key enzyme that renews intracellular lipids (phospholipids) and thereby affects the permeability of the outer membrane, the functioning of various membrane-bound proteins, and is also one of the pathogenic factors of Gram-negative bacteria [14]. It has a molecular weight of 31 kDa. The PldA molecule is a β-barrel formed by a β-sheet, consisting of 12 antiparallel amphiphilic β-strands. In an active form, PldA is a homodimer [14]. 

IBs are of interest not only as a potential source of a functionally active recombinant protein, but also as a model system for studying protein aggregation associated with so-called conformational diseases, including numerous neurodegenerative disorders and amyloidosis [15]. Information about the mechanisms of formation and disaggregation of IBs, as well as their molecular organization, is important for understanding the molecular basis of conformational diseases, and in the design of drugs for inhibiting or reversing protein aggregation. However, such data on IBs are limited. This fact is largely determined by the methodological difficulties of studying these conformationally heterogeneous aggregates. 

Green fluorescent protein (GFP) and GFP-like proteins were shown to be unique genetically encoded fluorescent markers for basic and applied research in the field of molecular and cellular biology. Currently, GFP are widely used to monitor the expression and folding quality of a recombinant protein under different bacterial culture conditions [6]. For this purpose, GFP is combined with the target protein so that the formation of a chromophore is possible only with the correct folding of the target protein [16,17]. This approach significantly simplifies the process of searching for the expression conditions of a recombinant protein in the native conformation. 

The aim of our work was to study the structure and properties of phospholipase A_1_ IBs formed at the optimal expression temperature in terms of their conformational quality, using a GFP tag as an indicator of recombinant protein folding. The research was focused on the following specific objectives: studying the effect of expression temperature on the conformational quality of PldA-GFP IBs; evaluating the secondary structure and arrangement of protein molecules inside the IBs; and collecting information about the stability and behavior of IBs in denaturing agents, as well as their enzymatic activity and interaction with eukaryotic cells.

## 2. Results and Discussion

### 2.1. Effect of the Cultivation Temperature Downshift on the Expression of the PldA-GFP Fusion Protein

To synthesize the PldA-GFP fusion, we have constructed the recombinant plasmid containing sequences of *pldA* encoding *Y. pseudotuberculosis* membrane bound phospholipase A_1_ and *gfp* encoding CopGFP based on pRSETa vector (Figure S1). The expression of the PldA-GFP at different cultivation temperatures (37, 26, and 18 °C) was induced by adding 0.1–1 mM IPTG followed by incubation for 3, 5 and 16 h. The amount of recombinant protein (based on intensity of the protein band on the SDS-PAGE) and the level of GFP fluorescence were determined for each experiment.

**Figure 1:**
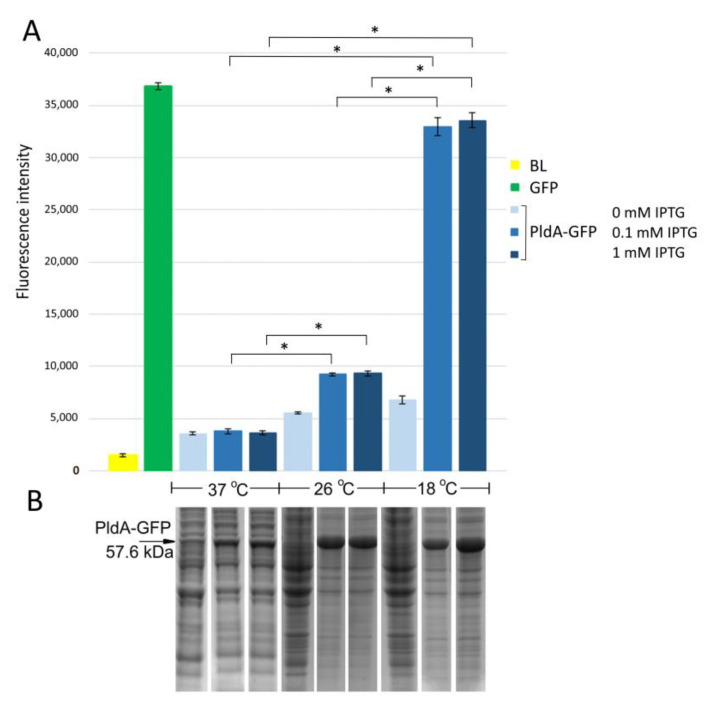
The level of GFP fluorescence in *E. coli* cells (**A**) and the expression of PldA-GFP fusion protein (**B**) in *E. coli* cell lysates after 16 h of incubation at different cultivation temperatures and IPTG concentrations. BL—plasmidless cells (negative control); GFP—cells expressing GFP protein (positive control). Fluorescence and bacterial mass for cell lysates were normalized according to OD_600_. The experiments were performed in three biological replicates. Results show the mean ± standard deviation (SD). The asterisk (*) indicates a significant difference (*p*-value < 0.05) in the fluorescence level between indicated groups.

*E. coli* strain BL21(DE3) pLysS/PldA-GFP expressed the major protein in the region of 57 kDa corresponding to the calculated molecular weight of the expected PldA-GFP fusion protein. It was found that lowering the cultivation temperature from 37 to 26 °C significantly increased the recombinant protein production (Figure 1B). A further decrease in the growth temperature to 18 °C did not have a noticeable effect on the level of protein synthesis. The maximum GFP fluorescence was observed in *E. coli* grown at 18 °C, it decreased by almost four times during bacterial cultivation at 26 °C, and was close to the negative control—plasmidless cell level at 37 °C (Figure 1A). Such effects of cultivation temperature on the level of PldA-GFP expression and GFP fluorescence were observed at all studied time points (data not shown), while the IPTG concentration did not significantly change the level of recombinant protein synthesis and GFP fluorescence of the fusion protein (Figure 1).

Thus, the low cultivation temperature promoted an increase in GFP fluorescence in the chimeric protein and, consequently, an increase in the proportion of the correctly folded form of phospholipase A_1_.

**Figure 2:**
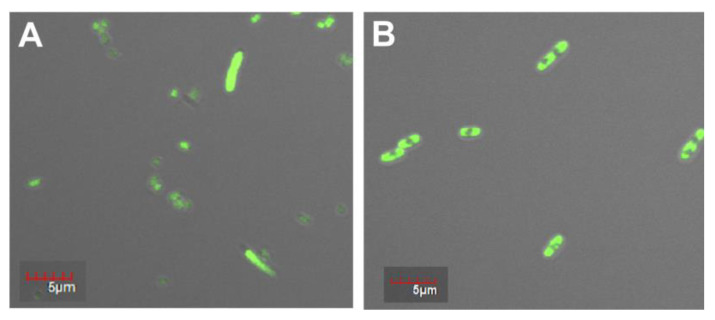
Fluorescence microscopy images of *E. coli* cells expressing GFP (**A**) and PldA-GFP (**B**).

**Figure 3:**
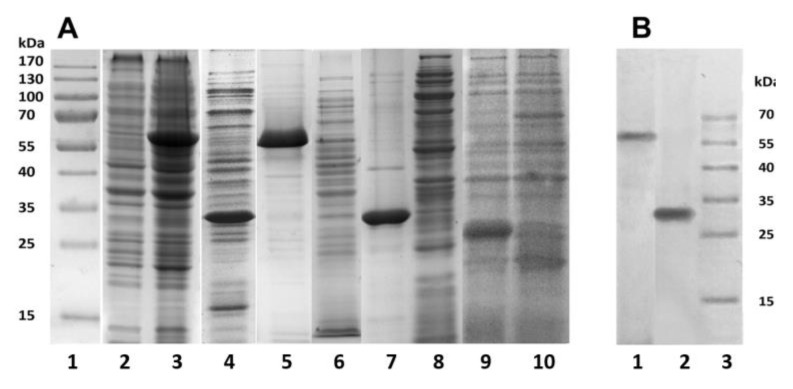
SDS-PAG electrophoresis (**A**) and Western blotting (**B**). (**A**): Whole cell lysates of *E. coli* BL plasmidless (2) and *E. coli* expressing PldA-GFP (3) and PldA (4); purified PldA-GFP IBs: pellet (5) and supernatant (6); purified PldA IBs: pellet (7) and supernatant (8); GFP: supernatant (9) and pellet (10); molecular weight standard (1). (**B**): PldA-GFP IBs (1); PldA IBs (positive control) (2); molecular weight standard (3). Western blotting was performed using mouse polyclonal antiserum to recombinant PldA.

Fluorescence confocal microscopy was used to study the morphology of the recombinant protein PldA-GFP and its localization in the cell. Figure 2 shows that homogeneous fluorescence throughout the cell cytoplasm was observed for bacteria expressing GFP only (Figure 3A), which is characteristic of the GFP soluble form. At the same time, under the similar culture conditions, the cells containing the pRSETa/PldA-GFP plasmid accumulated the chimeric protein in the form of aggregates (inclusion bodies), as evidenced by inhomogeneous fluorescence (Figure 2B). A similar morphology of inclusion bodies formed by chimeric proteins with GFP and other fluorophores, or mutant GFPs prone to IB formation, has been described in several works [3,18,19].

### 2.2. Isolation and Purification of PldA-GFP IBs, PldA IBs, and GFP

The proteins PldA-GFP, PldA, and GFP were expressed in *E. coli* at 18 °C, IPTG concentration of 0.1 mM, and cultivation time of 16 h (Figure 1). The bacterial cells were destroyed by lysozyme in combination with ultrasound, and the resulting suspensions were centrifuged. Using SDS-PAGE analysis, the recombinant proteins PldA-GFP and PldA were found only in insoluble fractions (pellets), while GFP was detected only in the supernatant (Figure 3A). These results confirm that in *E. coli* cells under mentioned expression conditions the chimeric protein and phospholipase A_1_ form IBs, while GFP is in a soluble form.

PldA-GFP IBs and PldA IBs were purified from impurities by sequential treatment with detergent solutions: 0.1% Sarcosyl and 1% sodium deoxycholate to remove LPS [20]. Denaturing SDS-PAGE showed that PldA-GFP and PldA migrate in the gel as bands corresponding to proteins with molecular weights of 57 and 31 kDa, respectively (Figure 3A). Both IBs samples barely contain any concomitant proteins. It should be noted that PldA-GFP loses GFP fluorescence under denaturing (2% SDS, boiled) and semi-native (0.1% SDS without boiled) SDS-PAGE. Immunoblotting using antibodies to PldA found the only protein band with MW of 57 kDa, which belongs to PldA-GFP (Figure 3B).

Recombinant GFP (rGFP) was isolated from the supernatant obtained by centrifugation of the lysed *E. coli* cells under the conditions described above using gel chromatography on a Superdex 200HR column. The fluorescent fractions were pooled and considered as containing GFP in its native conformation, as the formation of a fluorophore occurs spontaneously after this protein folding [21].

According to gel filtration data, GFP in PBS (pH 7.4) has an apparent molecular weight of approximately 42 kDa and, therefore, exists under these conditions as a dimer. Under denaturing (2% SDS and boiling) and semi-native SDS-PAGE (0.1% SDS without boiling), GFP migrates as a monomer with an apparent molecular weight of about 26 kDa, and loses fluorescence (Figure 3). Therefore, purified GFP is a dimer that has a fluorescence spectrum with maxima ex/em at 482/502 nm, and may be converted to a non-fluorescent monomer in the presence of a denaturant (SDS).

**Figure 4:**
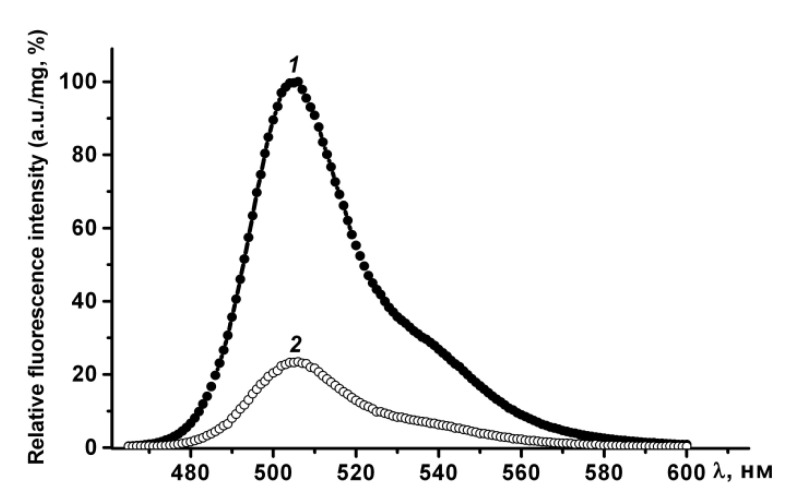
GFP fluorescence emission spectra of recombinant proteins in PBS: (1) GFP, (2) PldA-GFP IBs. The fluorescence intensity of GFP calculated per 1 mg of protein was taken as 100%.

In phosphate-buffered saline PldA-GFP IBs have the same fluorescence spectrum as rGFP, and therefore contain the recombinant protein in a native-like conformation. The fluorescence intensity of IBs, calculated per mg of GFP, was four times lower than that of GFP (Figure 4). Therefore, in IBs only 24, 5%, of the chimeric protein is in a fluorescently active form. However, this value is not entirely correct. It can be either overestimated, as according to CD spectroscopy data, recombinant GFP contains a certain amount of an inactive protein; and underestimated, as is known, the aggregation of GFP leads to the loss of a large part of its fluorescent activity [22]. 

### 2.3. Stability of PldA-GFP IBs

**Figure 5:**
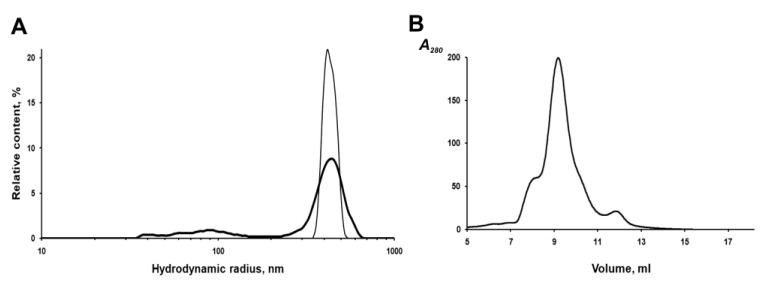
Characteristic of PldA-GFP IBs. (**A**) Particle size volume distribution of IBs in PBS (20 μg/mL): IBs intact (thin line), IBs after passing through a 0.1 mm needle and sonication (thick line); (**B**) Gel filtration chromatography of PldA-GFP solubilized in 0.1% SDS, 50 mM Tris-HCl buffer, pH 8.0, on a Superdex 200 HR column.

The IBs of the chimeric protein are rather stable in aqueous suspensions. According to the dynamic light scattering (DLS) data, IBs have a monomodal size distribution with an average hydrodynamic radius (R_H_) of 412 nm in PBS at a concentration of 2 to 20 μg/mL (Figure 5A). However, repeated passing of the IBs suspension (20 μg/mL) through a 0.1 mm needle, followed by sonication to homogenize aggregates in PBS, leads to the appearance of a population of small particles (40–100 nm), which account for about 18% of the total volume, and a decrease in the average IBs radius (by volume) to 367 nm (Figure 5A). As was reported earlier, sonication can disturb the structure of IBs and leads to the loss of some protein from their surface and an increase in porosity [23].

Stability in solutions of denaturants is an important characteristic of IBs, as it is directly related to their structural organization and conformation of the protein which forms them. As is known, IBs with the high protein content in native or native-like conformation are looser and more unstable than IBs, where the protein is mainly in the form of partially or improperly folded intermediates. They are usually dissolved in mild detergents and solutions with a low molar concentration of chaotropic agents [24]. The investigation of the stability of PldA-GFP IBs in SDS (0.01–0.1%) and urea (1–8 M) was carried out using the method of turbidimetry and GFP fluorescence.

**Figure 6:**
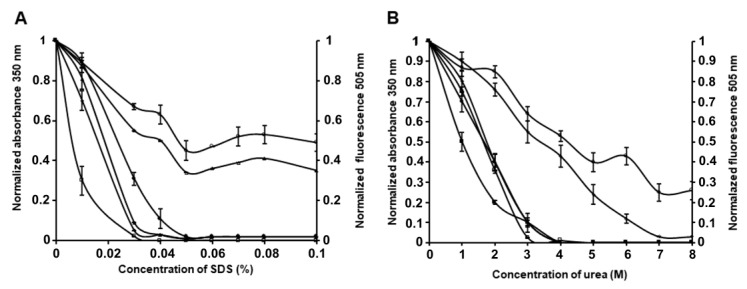
Solubility of PldA-GFP IBs in SDS (**A**) and urea (**B**). Dependence of turbidity signal (solid symbols) and GFP fluorescence (empty symbols) of IBs incubated in denaturant solutions for 5 min (circle), 1 h (triangle), 2 h (rhombus), and 24 h (square) on the denaturant concentration. The experiments were performed in three biological replicates. Results show the mean ± standard deviation (SD).

As we can see from Figure 6A, the optical density at 350 nm of IBs solutions drops sharply with an increase in the concentration of the detergent from 0.02 to 0.03%, indicating a reduction in the number of large particles, and reaches zero at 0.05% SDS.

Simultaneously with the solubilization of IBs, their GFP fluorescence decreases. In 0.05% SDS, the fluorescence intensity of IBs is 47% of the initial value, and remains at this level for 60 min with an increase in the concentration of the detergent to 0.1%. Increasing the incubation time to 2 h leads to the GFP fluorescence of solubilized IBs falling to a low value in 0.03% and being completely absent in 0.05% SDS. 

The results obtained show that the correctly folded chimeric protein, which is present in IBs, interacts with denaturant differently. One portion of the PldA-GFP fusion protein is solubilized from IBs with a simultaneous its denaturation and loss of activity, while the other portion goes into solution, retaining the native-like conformation and the fluorescent properties. This fact may be a consequence of the fluorescently active protein different localization within IBs [25] and, therefore, the availability for the detergent, as well as differences in its macromolecular and/or supramolecular structure. The latter assumption may be supported by gel chromatography data below about the structure of the fluorescent chimeric protein.

To characterize the fluorescently active protein, IBs were solubilized in 0.1% SDS and fractionated on a Superdex 200HR column. As shown in Figure 5B, fluorescent PldA-GFP was eluted from the column as three peaks, one major and two minor, corresponding to proteins with apparent molecular weights of ~600, 423 (major), and 120 kDa. As shown above, active GFP obtained by gel chromatography on Superdex 200HR is a dimer. Based on the data of gel chromatography, we can suppose that the fluorescent chimeric protein is a dimer (MW 114 kDa), which in 0.1% SDS exists mainly as an oligomer consisting of four dimers. It is possible that correctly folded PldA-GFP molecules are capable of forming more densely packed oligomeric structures in IBs and, therefore, more resistant to denaturants than the rest of the aggregate. As is known, the quaternary structure is an important factor of the conformational stability of fluorescent proteins [26].

The stability of PldA-GFP IBs in urea was also determined (Figure 6B). The turbidity of the IBs suspension decreases with an increase in the concentration of urea and the incubation time and drops to zero at a chaotropic agent concentration of 4 M. At the same time, in 4 M urea, the protein retains its fluorescence at the level of 53, 43, and 1.2% of the original value after incubation for 5 min, 2 h, and 24 h, respectively. These data are consistent with the findings of the study of IBs solubility in SDS. At the same time, urea is apparently a milder denaturing agent regarding the recombinant protein than SDS, as in 4 M urea, compared to 0.05% SDS (the minimum concentrations of denaturants providing, according to the data turbidimetry, complete dissolution of IBs), the recombinant protein retains its activity for a longer time. The retention of fluorescent activity by the recombinant protein solubilized from IBs with a short exposure in 0.05% SDS and 4 M urea suggests that the intermolecular interactions stabilizing IBs are less resistant to the denaturant than intramolecular contacts that provide the native conformation and activity of GFP. These results suggest that it is possible to select such solubilization conditions when the network of intermolecular contacts in IBs will be specifically disrupted, without denaturation of the correctly folded protein incorporated into the aggregates. As was demonstrated, maintaining a native-like secondary structure of the protein during its solubilization from IBs increases the yield of the recombinant protein during its refolding [27].

### 2.4. The Proteinase K Digestion of the IBs

**Figure 7:**
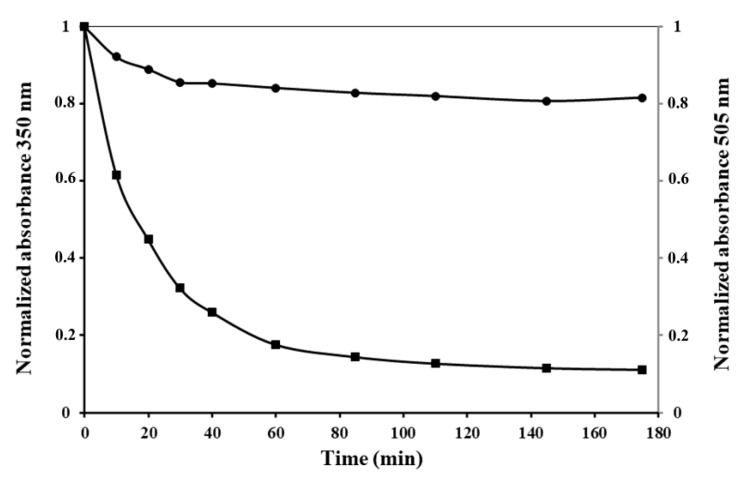
Proteinase K digestion of PldA-GFP IBs. Profiles of IBs fluorescence (circle) and turbidity (square).

PldA-GFP IBs were treated with proteinase K. Kinetics of proteolytic digestion of IBs was monitored by the measurement of turbidity at 350 nm and GFP fluorescence emission of the suspension over time. As it is shown in Figure 7, the turbidity of the IBs suspension decreases to its lowest level after 30 min of incubation, while the GFP fluorescence of the fusion protein remains practically unchanged.

The results presented here strongly suggest that more than one chimeric protein species with different protease resistance coexist within IBs. Non-fluorescent species of the recombinant protein, which are highly sensitive to proteolysis, appear to be misfolded or partially folded polypeptides. Proteinase K showed a strong preference for hydrolyzing unstructured protein regions [28].

The resistance of the active protein to proteinase K digestion suggests that it has a compact supramolecular structure, as the quaternary structure of GFP has been shown to be highly resistant to proteases [26]. However, it is also possible that proteinase K partially cleaves active protein, including GFP, without affecting the chromophore. As is known, the intensity of fluorescence of the chromophore can be changed insignificantly with major disturbances of the native structure of GFP [29]. The obtained data show the structural heterogeneity of PldA-GFP IBs. It can be assumed that compactly packed, properly folded, proteolytic resistant, and structurally less organized polypeptides susceptible to proteolysis can coexist in PldA-GFP IBs. 

### 2.5. CD Spectroscopy

**Figure 8:**
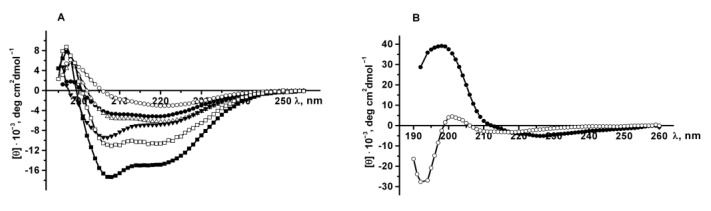
Far UV CD spectra of PldA-GFP, PldA and GFP. (**A**) PldA-GFP (●), GFP (Δ), and PldA (■): 0.03% SDS, 10 min; PldA-GFP (▼) and GFP (□): 0.1% SDS, 2 h; GFP: PBS (○); (**B**) PldA-GFP: 4 M urea, 2 h (●) and 24 h (○).

The secondary structure of the recombinant proteins was determined by CD spectroscopy in the far ultraviolet region. CD spectra of PldA-GFP, PldA and GFP in 0.03% SDS (exposure time from 10 min to 2 h and 24 h) and in 0.1% SDS (incubation time 2 and 24 h), as well as the spectrum of GFP in PBS were obtained (Figure 8A). According to turbidimetry data (Figure 6A), in these solutions of the detergent, almost complete solubilization of the recombinant protein from IBs is observed.

Recombinant GFP in PBS has a CD spectrum with a maximum at 198 nm and only one minimum at 220 nm, characteristic of proteins with β-pleated sheet structure (Figure 8A). As shown earlier [30], the *Y. pseudotuberculosis* phospholipase A_1_, which, similar to GFP, has the cylindrical β-sheet structure, exhibits a CD spectrum with a positive band at 193 nm and a broad negative band centered at 215 nm. The CD spectra of the recombinant proteins PldA-GFP, PldA and GFP in SDS solutions are characterized by a maximum at 197 nm and two minima at 208–207 nm and 219–217 nm, and are typical for mixed α-β proteins. Increasing the SDS concentration and incubation time caused an increase in the spectra amplitude and a decrease in the peak ratio at 218 and 207 nm which indicates an increase in the content of the α-helix in the protein (Figure 8A).

Using the CDPro software [31] the content of secondary structure elements of the recombinant proteins was determined (Table 1).

As can be seen from Table 1, in 0.03% SDS PldA-GFP, PldA, and GFP have a pronounced secondary structure including α-helices and β-pleated sheets. However, the content of α-helices and random coil structure in these recombinant proteins is higher than in the corresponding native proteins. These data suggest that in the studied proteins, molecules with a native-like structure, which are fluorescently active, coexist with partially and misfolded polypeptides or segments. An increase in SDS concentration and incubation time in detergent solutions leads to an increase in the content of α-helices and a decrease in the content of β-structure. At the same time, the content of regular (α-helix and β-sheet) and random coil structure remains practically unchanged, and is about 50% and 28–32%, respectively. The data obtained suggest that SDS induces the β-sheet to α-helix structural conversion in the recombinant proteins. A similar type of denaturation by SDS has been found for some globular proteins that have a β-sheet structure [34]. In addition, it has been shown earlier that SDS-denatured PldA *E. coli* has a non-native secondary structure with a high α-helix content [35].

As follows from the data presented in Table 1, PldA-GFP and PldA, dissolved from IBs with SDS at the same conditions, differ significantly in the content of β-structure, which is 3–4 times lower in PldA than in the fusion protein. Based on these data, it can be assumed that PldA-moiety in the chimeric protein, as compared to PldA, has a secondary structure that is closer to the native one, and is more resistant to denaturation by SDS. Thus, the use of GFP as a fusion partner appears to improve the folding of PldA when expressed in *E. coli.*

Comparative analysis of the spectral data of GFP and PldA-GFP solubilized in 0.1% SDS during incubation for 2 and 24 h showed that the structural changes of GFP induced by SDS, compared with those of PldA-GFP, occur more slowly and lead to a more significant decrease in the content of β-structure in the protein (Figure 8, Table 1). As a result, we can assume that GFP is more sensitive to SDS-denaturation than GFP-moiety in chimeric protein.

The secondary structure of PldA-GFP solubilized from IBs with 4 M urea was also determined. The CD spectrum of the recombinant protein in urea (incubation time 2 h) has a maximum at 197 nm, minima at 225 and 218 nm, and is specific for proteins with a β-structure (Figure 8B). The long-wavelength position of the negative band (225 nm) in the CD spectrum is probably due to the aggregated state of the solubilized protein. An increase in exposure time of the IBs in urea solution to 24 h leads to the widening of the PldA-GFP spectrum with a decrease in the ellipticity of the positive and negative (−5294 to −3220 deg cm^2^ dmol^−1^) bands, a shift in the positions of the minima to 217 and 211 nm, and the appearance of an intense negative band at 193 nm, which indicates an increase in the content of random coil structures in the protein. A quantitative analysis of CD spectra of PldA-GFP indicates a decrease in the content of both α-helix (8.8 to 0.6%) and β-sheet (34.7 to 31.7%) with an increase in random coil structure fraction (33.8 to 45.5%), when the incubation time of protein in urea increases. We can assume that urea not only dissolves the recombinant protein from IBs, but also denatures it. It should be noted that denaturation of *E. coli* PldA in the presence of urea is accompanied by the formation of a random coil structure [35]. Thus, urea induces an increase in the content of random coil conformation in PldA-GFP, while maintaining a high percentage of β-sheet structure. The protein species with a native-like β-sheet structure appear to be more resistance to urea, than misfolded or partially folded polypeptides.

### 2.6. Interaction of PldA-GFP IBs with Amyloid-Specific Dye Thioflavin T

Recent studies have shown that recombinant protein in IBs can have, along with other conformations, the structure of an intermolecular β-sheet (cross-β conformation), which is characteristic of amyloid proto-fibrils and fibrils [36]. As is known, the bacterial outer membrane proteins with the β-pleated sheet secondary structure have a high propensity to form amyloid-like structures [37]. We have previously shown the presence of amyloids in PldA IBs formed in *E. coli* at 37 °C [33].

A specific fluorescent dye, Thioflavin T (ThT), was used for the detection of amyloids in PldA-GFP IBs [38]. It should be noted that ThT allows detecting the amyloids in IBs in the presence of a protein with the native β-sheet structure, i.e., to differentiate the β-sheet conformation of the native protein from the structure of an intermolecular β-sheet in an aggregated form (amyloid fibrils) of the same protein [39]. This is important in the present study as the native-like structure of PldA is a β-sheet.

**Figure 9:**
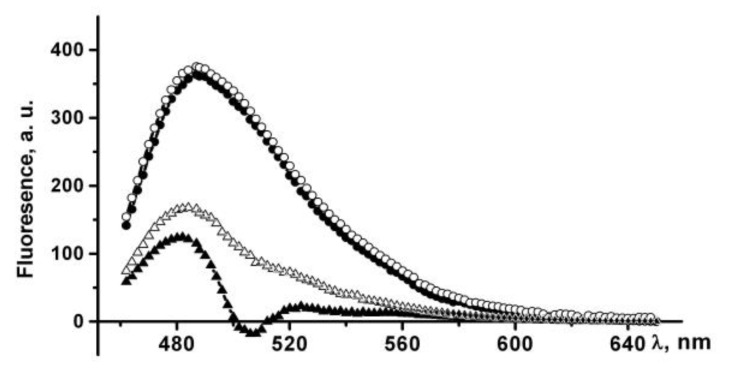
Difference fluorescence spectra of Thioflavin T bound to PldA-GFP IBs. IBs were incubated in PBS (triangle) and 0.04% SDS (circle) for 2 h (solid symbols) and 24 h (empty symbols).

The ThT fluorescence intensity at 484 nm increases by more than 100 times in the presence of PldA-GFP IBs suspended in PBS, which indicates the existence of amyloid-like structures in IBs. An increase in the incubation time of IBs in PBS leads to a slight increase in the dye fluorescence intensity (Figure 9).

IBs treated with 0.04% SDS also bind ThT, which is accompanied by a 300-fold increase in the dye fluorescence intensity (Figure 9). The incubation time of IBs in a detergent solution (10 min and 24 h) does not affect the fluorescence intensity of a bound ThT. A three-fold enhancement in the fluorescence of ThT in the presence of IBs incubated in SDS relative to untreated IBs may be due to several reasons: an increase in both the content of amyloids in IBs and their availability for dye binding, or changes in the structural and physical characteristics of the amyloids. The supposed changes in the amyloids in detergent-treated IBs seem to be quite realistic, as IBs solubilization occurs in the presence of SDS, and it has also been shown that SDS can induce the formation and modification of amyloid-like structures [40,41].

### 2.7. Enzymatic Activity of Phospholipase A_1_ Associated with and Released from IBs

As is known, IBs with a high protein content in the native or native-like conformation are easily soluble in the presence of relatively low molar concentrations of chaotropic agents [24,42]. The chimeric protein was solubilized from PldA-GFP IBs in 4 M urea. As shown above, the efficiency of IBs solubilization under these conditions was rather high, and the isolated recombinant protein contained a high percentage of β-structure. Parallel to this, IBs were dissolved under extremely harsh conditions in a traditional manner using high concentration of denaturant—8 M urea. The specific enzymatic activity of the PldA-GFP IBs suspended in a Tris-HCl buffer solution and the isolated recombinant protein solubilized in urea was determined after their incubation in the presence of Triton X-100 micelles, which is necessary for the formation of a functionally active spatial structure of PldA [35].

No studied samples of isolated PldA-GFP fusion protein and IBs exhibited enzymatic activity. The lack of phospholipase A_1_ activity is probably due to oligomerization of GFP, as it is known that the monomeric state of GFP is a necessary condition for maximum preservation of the native function of the target protein [43]. In addition, the properties of the linker sequence (flexibility, length, hydrophobicity, etc.) could affect the conformation and activity of the fusion protein [22].

To confirm the GFP inhibitory effect on the fusion protein enzymatic activity, PldA was expressed in *E. coli* at 18 °C and PldA IBs were isolated and purified. The PldA IBs suspended in a Tris-HCl buffer solution showed an enzymatic activity of 5 μmol/min per 1 mg. The detected IBs enzymatic activity despite its low level demonstrates the fundamental possibility of obtaining functionally active PldA IBs. The PldA, which was solubilized from IBs with 4 M urea, had a 5-fold higher activity (25–30 μmol/min per 1 mg). Attention is drawn to the fact that the recombinant protein in the functionally active form was obtained by passing the stage of complete unfolding in 8 M urea. It should be noted that PldA IBs expressed at 37 °C were inactive [33].

### 2.8. Interaction of PldA-GFP IBs with Neuroblastoma Cells

**Figure 10:**
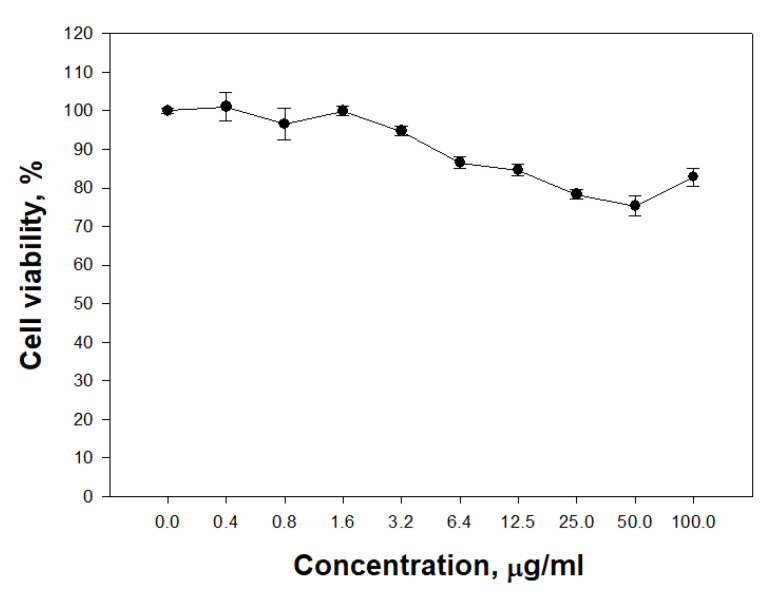
Cytotoxic activity of PldA-GFP IBs on mouse neuroblastoma Neuro-2a cells. The data are presented as the mean ± SEM values (*n* = 3).

The ability of PldA-GFP IBs to penetrate eukaryotic cells was studied. It was found that IBs of the chimeric protein is not toxic to neuronal cells in the range of concentrations from 0.4 to 100 μg/mL (Figure 10). 

**Figure 11:**
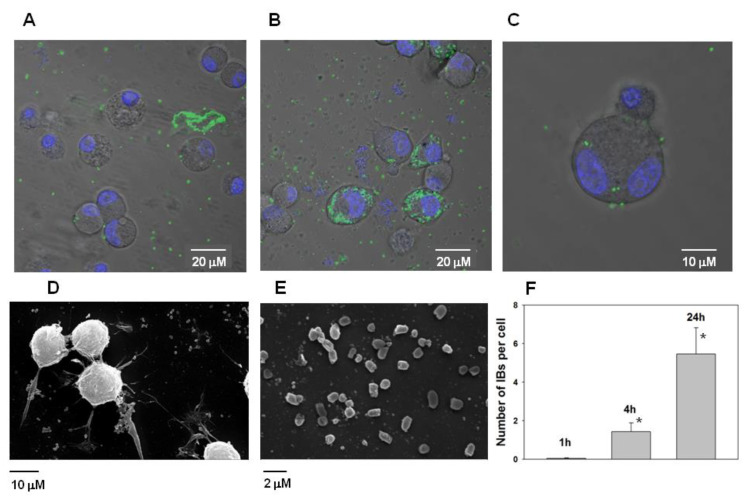
Fluorescence image of mouse neuroblastoma Neuro-2a cells after incubation with PldA-GFP IBs (20 μg/mL) for 4 (**A**) and 24 h (**B**). Images are obtained by superimposing the passing and fluorescent channels. Fluorescence of IBs is shown in green (GFP), cell nuclei are shown in blue (Hoechst 33342). An enlarged image (×5) of a neuroblastoma cell with included PldA-GFP IBs inside (**C**); Scanning electron microscopy of Neuro-2a mouse neuroblastoma cells with adsorbed PldA-GFP IBs (**D**); and magnified image (×5) of PldA-GFP IBs alone (**E**). The average amount of PldA-GFP IBs in a single neuroblastoma Neuro-2a cell after incubation for 1, 4, and 24 h (**F**). The fluorescent images of 150 randomly selected cells for each time point were inspected, and the amount of fluorescent IBs penetrating the cell surface was calculated and expressed as number of PldA-GFP IBs per cell. The data are presented as the mean ± SEM values (*n* = 150); * *p* < 0.05 compared with 1 h.

However, it was found that IBs have the property to bind the neuroblastoma Neuro-2a cells and subsequently penetrate them (Figure 11A–C). According the analysis of electron microscopic images of Neuro-2a cells and PldA-GFP IBs, the size of the IBs of the chimeric protein is in the range of 800–1000 nm (Figure 11D,E).

According to fluorescence image analysis, the average amount of PldA-GFP IBs penetrating the neuroblastoma Neuro-2a cell in control (0 h) is not detectable (data not shown), in 1 h is 0.05 ± 0.03, in 4 h is 1.4 ± 0.5, while in 24 h this value increases almost 5 times to 5.5 ± 1.4 IBs particles per cell in comparison with 1 h (Figure 11F). 

In summary, phospholipase A_1_ fused with GFP was expressed at different growth temperatures (37, 26, and 18 °C). The conformational quality of PldA-GFP IBs, as reflected by its specific fluorescence emission, enhanced by producing them at low expression temperatures. In this regard, the membrane beta-barrel protein phospholipase A_1_ is similar to most soluble globular proteins. The maximum content of the fusion protein in the native-like conformation was found in IBs produced at 18 °C. We studied the size, stability in denaturant solutions, and enzymatic and biological activity of these IBs, and the secondary structure of the recombinant protein forming them.

PldA-GFP IBs have fluorescent activity which indicates the presence of the recombinant protein in a native-like conformation. According to the CD spectroscopy data, isolated PldA-GFP has a high β-sheet content. Amyloid-like structures were also found in the studied IBs. Solubilization of the chimeric protein from IBs with urea and SDS is accompanied by a modification of its secondary structure. The nature and degree of structural changes in the protein induced by these denaturants depends on the nature of the denaturant, its concentration, and incubation time of IBs. It was found that intermolecular interactions holding properly folded PldA-GFP molecules in IBs may be less resistant to denaturant action than intramolecular ones, which provide the spatial structure of the protein. This suggests that the recombinant protein can be released from IBs while maintaining the native-like conformation when using mild conditions for its solubilization.

The presence of a fluorescent marker in a properly folded chimeric protein provided additional information on the molecular organization of IBs. According to the data obtained, PldA-GFP IBs are structurally heterogeneous: compactly packed, properly folded, proteolytic resistant and structurally less organized, more sensitive to the action of denaturants and proteinase K, protein molecules coexist in IBs. We also found differences in the structural organization of the fluorescently active polypeptides in IBs.

Comparative characteristics of PldA-GFP IBs and PldA IBs, produced in *E. coli* under the same conditions, showed that fusion with GFP enables more correct folding of PldA, but negatively affects its enzymatic activity. This fact is possibly due to the GFP type and the linker structure, used in these experiments. At the same time, IBs formed by PldA at 18 °C had enzymatic activity. It was firstly found that a membrane protein embedded in IBs showed functional activity. As can be assumed from the data obtained, PldA fused with a monomeric type of GFP will form enzymatically active IBs at low temperatures; this remains to be clarified in our further studies.

Chimeric protein IBs is not toxic to eukaryotic cells and has the property of penetrating into neuroblastoma cells.

In the course of the work, it was shown that the GFP marker can be useful not only as an indicator of the target protein folding, but also as a tool for studying the structural organization of IBs, their morphology and localization in *E. coli* cells, as well as for visualizing IBs interaction with eukaryotic cells.

The culture of neuroblastoma cells was selected for experiments as eukaryotic cells do not have the ability to phagocytosis and, therefore, do not absorb PldA IBs in this way. In addition, Neuro-2a neuroblastoma cells are brain tumor cells. Our data confirm the possibility of delivering functionally active proteins to tumor cells using IBs, including for antitumor therapy.

## 3. Materials and Methods

### 3.1. Construction of Chimeric Insertion

The coding sequence of phospholipase PldA *Y. pseudotuberculosis* strain 488 (GenBank: MW848438) without signal sequence was amplified with primers: PldA-NdeI 5′-CCG**CATATG**GAAGCAACGATTGAAAAGATTC-3′ (forward) and PldA-Linker-R 5′-gttaattaaaccagcaccgtcaccAAGGACATCGTTCAACATGATAC-3′ (reverse). The pTurboGFP plasmid (Evrogen, Russia; GenBank: ASW25889.1) was used as a template for *gfp* amplification with primers: GFP-linker-F 5′-ggtgacggtgctggtttaattaacGCAGAAATCTATAACAAAGATGG-3′ (forward) and GFP-R 5′-TGAT**CTCGAG**TTATTCTTCACCGGCATCTGCATCCG-3′ (reverse). Bases in uppercase letters indicate *pldA* and *gfp* specific regions. Two restriction sites, *NdeI* and *XhoI*, (in bold letters) were incorporated at the 5′ and 3′ ends of the resulting insertion. In order to connect and improve the assembly of *pldA* and *gfp* we added the sequence coding flexible linker (GDGAGLIN) to the *pldA* reverse primer and the *gfp* forward primer (in lowercase letters). The gene fusion was assembled by II-rounded asymmetric PCR. At the first round, the *pldA* and *gfp* fragments were amplified in total volume of 20 µLeach. Reaction mixes included 250 µM of each dNTP, 0.2 U of Q5 High-Fidelity DNA Polymerase (Promega, Madison, WI, USA), 5× Q5 Reaction Buffer, 0.05 µM forward primer, and 0.5 µM reverse primer (for *pldA* production), or 0.5 µM forward primer and 0.05 µM reverse primer (for *gfp* production). The PCR conditions were as follows: initial denaturation at 95 °C for 5 min, then 21 cycles of 94 °C for 30 s, 55 °C for 30 s, and 72 °C for 40 s, followed by a final extension step at 72 °C for 2 min. In the second round, the reaction products were mixed, then an additional 0.2 U of Q5 High-Fidelity DNA Polymerase was added, and the combined reaction mix was amplified for 15 cycles at 94 °C for 30 s, 55 °C for 30 s, and 72 °C for 1 min, followed by a final extension step at 72 °C for 5 min. After amplification, the PCR fragment corresponding to the expected length of the chimeric insertion was purified and used for cloning.

### 3.2. PCR and Sequencing

PCR amplification of the *pldA* gene was performed using the primers PldA_NdeI 5′-CCG**CATATG**GAAGCAACGATTGAAAAGATTC-3′ and PldA_XhoI-Rev 5′-A**CTCGAG**TTAAAGGACATCGTTCAAC-3′, which included restriction sites for NdeI and *XhoI* (in bold letters). The expected amplicon size was 833 bp. PCR amplification of the *gfp* gene was performed using the primers GFP-F 5′-CCG**CATATG**GAGAGCGACGAGAGCGGCCTGCCCG-3′ and GFP-R 5′-TGAT**CTCGAG**TTATTCTTCACCGGCATCTGCATCCG-3′. The expected amplicon size was 715 bp. The PCR conditions were as follows: initial denaturation at 95 °C for 5 min, then 25 cycles of 94 °C for 15 s, 55 °C for 10 s, and 72 °C for 30 s, followed by a final extension step at 72 °C for 5 min. PCR-colonies was performed with standard T7-promoter/T7-terminator primers. The PCR fragments were evaluated on a 1.5% agarose gel stained with ethidium bromide. For subsequent cloning or sequencing, unincorporated primers and dNTPs were removed from PCR products with a GeneJET PCR Purification Kit (Thermo Fisher Scientific, Inc., Waltham, MA, USA). Sequencing was performed on a 3130xl Genetic Analyzer (Applied Biosystems; Thermo Fisher Scientific, Inc., Waltham, MA, USA) according to the manufacturer’s instructions.

### 3.3. Molecular Cloning

The plasmid vector pRSETa (Invitrogen; Thermo Fisher Scientific, Inc., Waltham, MA, USA), designed for gene expression in prokaryotic cells such as *E. coli* BL21 (DE3) pLysS strain, was used. Vector and inserts (pldA, gfp, and pldA-gfp) were digested by *NdeI* and *XhoI* restrictases (Thermo Scientific). Then, inserts were ligated into vectors. The ligated products were transformed into *E. coli* TOP10′F cells (Invitrogen) and the resultant transformants were selected on carbenicillin plates and subjected to PCR-colony with T7 promoter primers. Several of the PCR-positive clones were inoculated into 3 mL test-tube cultures and allowed to grow overnight at 37 °C in a shaker at 200 rpm. Then, plasmid DNA were extracted from these cultures with GeneJET Plasmid Miniprep Kit (Thermo Fisher Scientific Inc., Waltham, MA, USA). Appropriate insertions of genes into the pRSETa vector were verified by DNA sequencing.

### 3.4. Expression

For protein expression, pRSETa/pldA-GFP, pRSETa/mPldA, and pRSETa/GFP constructs were transformed into *E. coli* BL21(DE3)pLysS competent cells. Single colonies were picked and inoculated in 10 mL of LB broth containing carbenicillin (100 mg/mL) and chloramphenicol (35 mg/mL) and grown overnight at 37 °C with shaking at 200 rpm. After 12 h, 250 mL of LB broth containing the same antibiotics was inoculated with 2% (*v*/*v*) of overnight grown primary culture and the culture was kept in incubator set at 37 °C with constant shaking at 200 rpm. IPTG induction was conducted at different concentrations (from 0.1 to 1 mM) when culture OD_600_ reached 0.5–0.7. The culture was further maintained at different temperatures, 37, 26, and 18 °C, for 3, 5, and 16 h. Before and after induction, approximately equal numbers of cells from various cultures (normalized according to OD_600_ values) were lysed in sample buffer and analyzed by SDS–PAGE. The GFP fluorescence of the chimeric protein PldA-GFP in *E.coli* cells was measured on the FL-600 Fluorescence/Absorbance Plate Reader (Bio TEK Instruments, Winooski, VT, USA). Absorption measurements (OD_600_) and fluorescence intensity measurements (λexc. 488 nm/λexp. 530 nm) were made on 100 µLcell suspension. Plasmidless *E. coli* BL21(DE3) strain was used as negative control, and *E.coli* BL21(DE3) with pRSETa/GFP as positive control under all expression conditions. The *E. coli* BL21(DE3)pLysS/mPldA strain was expressed at 18 °C for 16 h.

All measurements were performed in three biological replicates. Results were expressed as the mean ± standard deviation (SD). The Student’s *t*-test was used to evaluate the data. Statistical significance was considered for *p*-values < 0.05.

### 3.5. Isolation of IBs and GFP

PldA-GFP IBs and PldA IBs were isolated as described in previous work [33]. Briefly, wet bacterial biomass was lysed by lysozyme and exposed to ultrasound. IBs were sedimented from the cell biomass lysate by centrifugation at 4500× *g* for 30 min. IBs pellets was sequentially treated with 10 mM Tris-HCl buffer (pH 8.0) containing 0.1% *N*-Lauroylsarcosine (Sarcosyl) and with the same buffer containing 1% sodium deoxycholate and 1 M urea for 15 min, with the aim to achieve a more thorough purification from contaminants (proteins, LPS, and nucleic acids). The protease inhibitor PMSF (1 mM) was used at all stages of IBs isolation. IBs obtained by this scheme were stored at −70 °C for a maximum of 30 days.

*E. coli* cells expressing GFP were lysed by lysozyme and exposed to ultrasound under the conditions described in previous work [33] and centrifugated at 4500× *g* for 30 min. GFP was isolated from supernatant by size-exclusion chromatography on a Superdex 200HR column in PBS with an elution rate of 0.5 mL/min using a FPLC system (Amersham Pharmacia Biotech).

### 3.6. Determination of PldA-GFP and GFP Oligomeric Structure

The molecular weight of GFP (in PBS) and PldA-GFP (in 0.1% SDS, 50 mM Tris-HCl buffer, pH 8.0) was estimated by size-exclusion chromatography on a Superdex 200 column 10/300 GL using a FPLC system (Amersham Pharmacia Biotech) with an elution rate of 0.5 mL/min. The column was calibrated using proteins with known molecular weights (“ICN Biomedicals”, Costa Mesa, CA, USA): apoferritin, bovine serum albumin, egg albumin, chymotrypsinogen, myoglobin, and cytochrome *c* (480, 67, 45, 24, 18, and 13 kDa, respectively). The relative error of the determination of the molecular weight was 5%.

### 3.7. Fluorescence Confocal Microscopy

Bacterial cells were pre-diluted in PBS to a concentration of 1 × 10^6^ cells/mL and 20 μL of this solution was placed between two circle cover slips (Thermo Scientific) with a thickness of 0.08 to 0.12 mm. GFP fluorescence was visualized using a confocal microscope assembled based on Olympus FV1200 system equipped with a 488 nm argon laser with EX DM 405/488 and gallium arsenide phosphide (GaAsP) detector with filter set (DM 570, BA 505–540) and a 100× objective lens (UAPON100XOTIRF, Olympus). Transmission channel was visualized using 635 nm diode laser and transmission light detector. All images were obtained using Olympus FLUOVIEW software v.4.1.a with 4 us/pix exposure, 1600 × 1600 resolution and 4 kalman for digital noise elimination.

The murine neuroblastoma cell line Neuro-2a (CCL-131^TM^) was purchased from American Type Culture Collection ATCC (ATCC). A cell suspension in DMEM medium (BioloT, Russia) containing 10% fetal bovine serum and 1% penicillin/streptomycin solution (BioloT, Russia) was introduced into the wells of chamber for confocal microscopy at a concentration of 1 × 10^3^ cells per well and left for adhesion for 24 h in a CO_2_-incubator (5% CO_2_, 37 °C). After that, a suspension of PldA-GFP IBs in PBS (20 μg/mL) was added to the cells and the chambers were additionally incubated for 0, 1, 4, and 24 h. The accumulation of IBs in Neuro-2a cells was studied using laser scanning confocal microscopy and scanning electron microscopy. The fluorescent images of 150 randomly selected cells for each time point were inspected and the amount of fluorescent IBs penetrating the cell surface was calculated and expressed as number of PldA-GFP IBs per cell.

Fluorescence images of PldA-GFP IBs and neuroblastoma Neuro-2a cells were obtained using an LSM 710 LIVE AxioObserver laser scanning confocal microscope (Carl Zeiss GmbH, Jena, Germany). Cell nuclei were stained using a Hoechst 33,342 fluorescent dye (Invitrogen, USA) at a concentration of 5 μM. Fluorescence was excited at 488 nm, and emission was recorded at 493–652 nm. Processing and subsequent analysis of cell images was performed using the ZEN 2011 software (Carl Zeiss GmbH, Jena, Germany).

### 3.8. Scanning Electron Microscopy

Neuroblastoma cells on cover slides were fixed in 4% (*v/v*) glutaraldehyde in 0.1 M phosphate buffer (pH 7.2) for 2 h at room temperature. After dehydrating the cells by passing them through an ethanol series, the ethanol was replaced by isoamyl acetate. Then, parts of the cover glass with cells were dried under Critical point dryers K850 equipment (Quorum Technologies, London, UK), mounted on stubs, and coated with carbon. The samples were examined at the Instrumental Centre of Biotechnology and Gene Engineering of FSCEATB FEB RAS using a Scanning electron microscope EVO (Carl Zeiss, Jena, Germany).

### 3.9. Cytotoxic Activity

The cytotoxic activity of PldA-GFP IBs was determined by MTT method. After incubation of mouse neuroblastoma Neuro-2a cells with IBs in 96-well microplate for 24 h at 37 °C and 5% CO_2_, the supernatant was replaced with pure medium. Then, 5 mg/mL MTT reagent solution (Sigma-Aldrich, St. Louis, MO, USA) was added to each well and microplate was incubated for additional 4 h, after which SDS-HCl solution (1 g SDS/10 mL dH_2_O/17 μL 6 N HCl) was added and incubated at 37 °C for 4–18 h. Absorption was measured at a wavelength of 570 nm using a Multiskan FC spectrophotometer (Thermo Scientific, Canada). The cytotoxic activity of IBs was expressed as the concentration of EC_50_ at which the metabolic activity of cells is inhibited by 50%.

### 3.10. Dynamic Light Scattering (DLS)

The size of PldA-GFP IBs was determined using the DLS technique with a ZetaSizer Nano ZS instrument (Malvern, UK) equipped with a He-Ne-laser (λ 633 nm, 4 mW) at 173° angle. The hydrodynamic radius (R_H_) of the protein particles was calculated using the software supplied with the instrument. IBs samples (0.1–0.6 mg/mL) were suspended in PBS, pH 7.5, by passing 10 times through a syringe microneedle and incubation at room temperature for a specified time with mixing. Data was accumulated for 5–60 min. Measurements were conducted in a 10 mm × 10 mm cuvette. Time of data accumulation for the correlation function was selected automatically via the instrument software, and it was 5–30 min. All measurements were replicated 2–3 times.

### 3.11. Solubility of PldA-GFP Ibs in Urea and SDS

IBs were suspended in 50 mM Tris-HCl buffer, pH 8.0, containing SDS from 0.01 to 0.1% or urea from 1 to 8 M, and incubated for a specified time (from 5 min to 24 h). Turbidity of the samples was measured at 350 nm with a μQuant spectrophotometer (BioTEK Instruments, Inc., USA). Fluorescence intensity of the PldA-GFP IBs solutions in urea and SDS was recorded with a FL-600 Fluorescence/Absorbance Plate Reader (BioTEK Instruments, USA) with excitation wavelength at 495 nm and emission at 530 nm. The experiments were performed in three biological replicates. Results show the mean ± standard deviation (SD).

### 3.12. Proteolytic Digestion of Inclusion Bodies

Purified PldA-GFP IBs were diluted to 1 OD at 350 nm in 980 mL of 50 mM Tris-HCl, 150 mM NaCl buffer of pH 8.0. Proteolytic digestion of IBs was initiated by adding 40 mL proteinase K (stock, 1 mg/mL) to the inclusion body solution (at 40 µg/mL final concentration). Proteolytic digestion was monitored for 120 min by measuring the changes in OD at 350 nm and in GFP fluorescence intensity (λexc. 488 nm/λexp. 530 nm).

### 3.13. The Thioflavin T Assay

To assess the presence of amyloid protein structure in IBs, we used the amyloid-specific dye Thioflavin T [34]. IBs were suspended in PBS (10 mM phosphate buffer, pH 7.4, containing 137 mM NaCl), or PBS containing 0.04% SDS, and incubated for a specified time (from 10 min to 24 h). The final reaction mixture for ThT assay consisted of 20 µg/mL protein and 20 µM ThT. The fluorescence measurements of the IBs samples were carried out at low SDS concentrations (0.004%) in order to avoid the influence of the detergent on the fluorescence intensity of ThT [44]. The samples were incubated for 10 min at room temperature. The fluorescence spectra were recorded in the range from 460 to 650 nm at λex = 440 nm. The background intensity from Raman scatter and ThT free samples were subtracted from each measurement sample of fluorescence intensity. Buffer controls did not show any significant ThT fluorescence.

### 3.14. Enzymatic Activity Measurement

The IB samples suspended in 50 mM Tris-HCl buffer (pH 8.0) or dissolved in the same buffer containing 4 or 8 M urea, were diluted 10 times with buffer solution (20 mM Tris-HCl, pH 8.3, 10 mM Triton X-100, 0.87 M urea) to a protein concentration of 10–40 μg/mL and incubated for 16 h at room temperature. The enzymatic activity of PldA was determined by the quantitative analysis of free oleic acid C18:1 formed during the hydrolysis of the substrate 1,2-dioleoyl-snglycero-3-phosphatidylcholine (Sigma-Aldrich, St. Louis, MO, USA) by GLC as described earlier [33]. The specific activity of PldA was expressed in μmol of the acid formed in 1 min per 1 mg of protein (IU/mg).

### 3.15. SDS-PAGE and Western Blotting

Whole cell-lysate proteins and recombinant proteins were separated by electrophoresis on 12% PAG under the standard denaturing conditions according to the method of Laemmli [45] with prior heating of the sample (5 min at 100 °C) in a buffer containing 2% SDS, as well as under non-denaturing conditions, using the method of semi-native PAGE [46]. In the latter case, the samples were dissolved at 0 °C in a buffer containing 0.1% SDS, without β-mercaptoethanol, and separated on a gel without SDS at 10 mA for 2.5 h at 5 °C. A set of colored proteins (Fermentas, Lithuania) with molecular weights of 10, 15, 25, 35, 40, 55, 70, 100, 130, and 170 kDa were used as markers. The proteins separated in the gel were stained with Coomassie R-250 in 10% acetic acid and 30% methanol. GFP fluorescence after SDS-PAGE was recorded using the VersaDoc imaging system (Bio-Rad, USA). The determination of the recombinant proteins molecular weight was carried out from the graph of the linear relationship between log MW of marker proteins (from 15 to 70 kDa) and relative migration distance (Rf) of these proteins according to the formula y = −0.0147x + 2.0684 (R2 > 0.994).

The localization of PldA in PldA-GFP IBs was assessed by Western blotting. After SDS-PAGE, proteins were transferred from non-stained gel on nitrocellulose membrane (0.2 μm, Merk Millipore, Burlington, MA, USA) by equipment for semi-dry transfer at the current of 0.8 mA/cm^2^ overnight at 4 °C according to the standard procedure [47]. Immunodetection was carried out by the protein detection system SNAP i.d. according to the instruction of the manufacturing company (Merk Millipore, Burlington, MA, USA). A murine polyclonal antiserum to recombinant PldA was prepared as described in [48]. HRP-Goat Anti-Mouse antibodies (Invitrogen, Waltham, MA, USA) were used according to the instruction of the manufacturing company. Antigen-antibody complexes were identified on nitrocellulose membrane by the hydrogen peroxide detection with 3,3′-diaminobenzidine for 20 min at the room temperature.

### 3.16. Spectroscopic Methods

#### 3.16.1. Circular Dichroism (CD)

*CD spectra* were recorded on Chirascan Plus CD spectrometer (Applied Photophysics, UK) in quartz cuvettes with an optical path length of 0.1 and 1 cm for the far-UV, or peptide for the near-UV or aromatic regions. The measurements were performed at room temperature (22 °C). Protein solutions in urea and detergent were centrifuged at 15,000× *g* for 20 min before taking the spectra. The presented data are the average of two independent measurements, each averaged of three scans. Each averaged CD spectrum was corrected for the buffer baseline by subtracting an averaged buffer CD signal over the same wavelength region. The content of the secondary structure elements in the recombinant proteins was calculated according to Sreerama method from software package CD*Pro* [31].

#### 3.16.2. UV-Spectra

Absorption spectra were recorded using a UV-Visible spectrophotometer UV-1601 PC (Shimadzu, Japan) in quartz cuvettes with an optical path length of 1 cm, 0.1 cm, 0.01 cm in the range from 190 to 400 nm. Protein concentration of GFP in the PBS and PldA and PldA-GFP in the urea and SDS solutions was determined (following centrifugation at 15,000× *g* for 20 min) from the UV spectra at absorption maximum of 280 nm assuming that the $A_{1\mathrm{cm}}^{0.1\%}$ value was 2.74—for *Y.pseudotuberculosis* phospholipase A_1_; 1. 36—for PldA-GFP and 1.34—for GFP.

#### 3.16.3. Fluorescence Spectra

The ThT and GFP fluorescence spectra were recorded on a RF-5301 PC spectrofluorophotometer (Shimadzu, Japan) in quartz cuvettes with an optical path length of 1 cm in the range from 460 to 650 nm at λex = 450 nm. The excitation and emission slit widths were set at 5 nm. The measurements of GFP fluorescence activity of the recombinant proteins were performed in the range of low concentrations (0.1–1.1 mg/mL), where the intensity of GFP fluorescence is directly proportional to its concentration. For comparison, all the GFP fluorescence data were normalized by the maximum fluorescence signal.

## 4. Conclusions

Despite their fundamental and practical importance, IBs are not well understood. To date, there are many unresolved and controversial issues regarding the conformational states and arrangement of protein molecules inside the IBs, as well as the exact nature of intermolecular interactions. Various biophysical methods have been used to characterize proteins aggregated in IBs, including NMR one, which enables detailed structural information at the specific residue level up to the three-dimensional structure of the protein to be obtained. However, no significant progress has been made in these studies so far.

Here, we used GFP as a fusion tag to provide further opportunities for structural studies of IBs. Therefore, some details of the inner organization IBs were revealed. The data obtained indicate the presence of highly structured clusters of correctly folded protein molecules inside the IBs, which can differ from one another in the degree of order in the structure. Intramolecular interactions that maintain the native-like conformation of the protein in these clusters may be more resistant to denaturants than intermolecular contacts in IBs.

According to our data, PldA-GFP IBs formed at low temperatures contain a noticeable percentage of the protein in the native conformation, and the recombinant protein solubilized from IBs has a high content of β-structure. This fact suggests that IB formation occurs at a relatively late stage of protein folding, when the degree of protein structural organization is high enough and proteins retain most of their secondary structure during aggregation. The results of the structural analysis of PldA-GFP IBs contribute to understanding the mechanisms of IB formation and their structure.

The accumulation of knowledge about IBs formed by proteins of different nature under various expression conditions provides a deeper understanding of their molecular organization, opening possibilities for controlling their structure and the process of formation. Further progress in the study of IBs will require new methodological approaches, as well as powerful biophysical research methods.

## Figures and Tables

**Table 1:** Content of secondary structure elements (%) in PldA-GFP, PldA and GFP, dissolved in SDS and PBS.

Sample	α-Helix, %	β-Sheet, %	β-Turns, %	Random coil, %
GFP, PBS	6.1	40.8	21.3	31.8
GFP, 0.03% SDS, 15 min	20.1	29.6	21.9	28.4
GFP, 0.1% SDS, 2 h	32.0	18.2	22.9	26.9
GFP, 0.1% SDS, 24 h	41.5	9.1	20.9	28.5
PldA-GFP, 0.1% SDS, 2 h	22.3	26.1	21.5	30.1
PldA-GFP, 0.1% SDS, 24 h	21.9	23.0	23.5	31.6
PldA-GFP, 0.03% SDS, 2 h	14.9	30.6	22.1	32.4
PldA, 0.03% SDS, 2 h	39.7	10.1	22.6	27.6
GFP, 0.01 M sodium phosphate, pH 7.5 [32]	20 ± 1	52 ± 2	16 ± 1	13 ± 1
PldA (OMPLA *E. coli*) *	7.7	61	11.9	19.2
PldA of *Y. pseudotuberculosis* (predicted secondary structure) [33]	6.5	56.7	29.0	7.8

* UniProtKB—POA921 (PA1-*E.coli*).

## Data Availability

Data is contained within the article and supplementary material.

## Supplementary Materials

The following are available online: Figure S1. Recombinant plasmid for the expression of the PldA-GFP fusion protein.

## Sample Availability

Samples of the compounds are available by agreement with the authors and management of the organization for conducting joint research from the authors.

## Abbreviations

PldA—detergent-resistant phospholipase A_1_; GFP—green fluorescent protein; PldA-GFP—chimeric protein; IB—inclusion body; CD—circular dichroism; DLS—dynamic light scattering; LPS—lipopolysaccharide; SDS-PAGE—sodium dodecyl-sulfate polyacrylamide gel electrophoresis; ThT—Thioflavin T; R_H_—hydrodynamic radius; PBS—phosphate-buffered saline, pH 7.5; and IPTG—isopropyl β-D-1-thiogalactopyranoside.
