# Supplementary material for: Studies on the Structure and Properties of Membrane Phospholipase A1 Inclusion Bodies Formed at Low Growth Temperatures Using GFP Fusion Strategy
Source: Molecules. 2021 Jun 28;26(13):3936. doi: 10.3390/molecules26133936 (PMC8271855; doi:10.3390/molecules26133936)
Supplement: Supplementary file 1 [file molecules-26-03936-s001.zip › molecules-1226874-supplementary.pdf]

Supplementary Materials

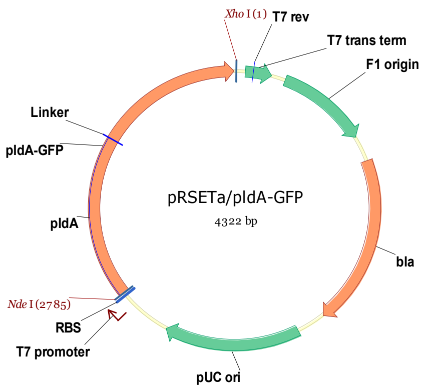

Figure 1. Recombinant plasmid for the expression of the PldA-GFP fusion protein.
